# Conditional Enhanced Variational Autoencoder-Heterogeneous Graph Attention Neural Network: A Novel Fault Diagnosis Method for Electric Rudders Based on Heterogeneous Information

**DOI:** 10.3390/s24010272

**Published:** 2024-01-02

**Authors:** Ximing Cao, Ruifeng Yang, Chenxia Guo, Hao Qin

**Affiliations:** 1 School of Instrument and Electronics, North University of China, Taiyuan 030051, China; b20230625@st.nuc.edu.cn (X.C.); guochenxia@nuc.edu.cn (C.G.); 2 School of Instrumentation and Optoelectronic Engineering, Beihang University, Beijing 100191, China; qhbuaa@buaa.edu.cn

**Keywords:** fault diagnosis, graph data, electric rudder, prognostics and health management

## Abstract

In machine fault diagnosis, despite the wealth of information multi-sensor data provide for constructing high-quality graphs, existing graph data-driven diagnostic methods face challenges posed by handling these heterogeneous multi-sensor data. To address this issue, we propose CEVAE-HGANN, an innovative model for fault diagnosis based on the electric rudder, which can process heterogeneous data efficiently. Initially, we facilitate interaction between conditional information and the original features, followed by dimensional reduction via a conditional enhanced variational autoencoder, thereby achieving a more robust state representation. Subsequently, we define two meta-paths and employ both the Euclidean distance and Pearson coefficient in crafting an effective adjacency matrix to delineate the relationships among edges within the graph, thereby effectively representing the complex interrelations among these subsystems. Ultimately, we incorporate heterogeneous graph attention neural networks for classification, which emphasizes the connections among different subsystems, moving beyond the reliance on node-level fault identification and effectively capturing the complex interactions between subsystems. The experimental outcomes substantiate the superiority of the electric rudder-based CEVAE-HGANN model fault diagnosis.

## 1. Introduction

Electric rudders (ERs) are extensively utilized in industrial control areas requiring high precision, particularly in the attitude transition of hypersonic aircraft. The Prognostics and Health Management (PHM) system has been proposed to mitigate the potential damage caused by ER faults [1]. Among them, the fault diagnosis (FD) component within the PHM is crucial for maintaining the reliability and safety of ERs.

Driven by advancements in artificial intelligence and the Internet of Things, the evolution of signal acquisition and analysis technologies has facilitated the collection of extensive industrial data, which has accelerated the improvement of data-driven FD methodologies. Data structures in ER-based, data-driven FD methodologies can be broadly classified as Euclidean or graph-structured. Machine learning [2,3,4] and deep learning [5,6,7] Euclidean data-based methods have led to significant achievements in ER-based, data-driven FD methods. However, Euclidean data present certain limitations in some complex application scenarios, especially in terms of interactive data in multiple systems [8]. In complex FD scenarios, researchers have explored additional methods to overcome the limitations of Euclidean data, such as multimodal and transfer learning. Multimodal learning, which integrates data from various modalities, compensates for the inadequacies of singular Euclidean data sources in capturing complex, multidimensional, and dynamic relationships [9,10,11]. Transfer learning enhances a model’s adaptability and performance in new domains by transferring knowledge between different but related tasks or fields [12,13]. Although transfer learning does not directly address the constraints of Euclidean data structures, it indirectly compensates for them by enhancing a model’s ability to process various data sources, thus mitigating the limitations of Euclidean data-based models in capturing complex system characteristics.

Although researchers have made progress in complex scenarios using the above methods, these methods still rely on traditional Euclidean data representations. Conversely, graph-structured data offer a more flexible approach to depicting complex relationships between data points and facilitate exploring intricate systems using FD methods. Among them, Graph Neural Networks (GNNs) have attracted attention in graph information data mining due to their powerful inference capabilities and interpretability. Li et al. [14] proposed a Multi-Receptive Field Graph Convolutional Network (MRF-GCN), pioneering the application of GNNs to mechanical FD. In [15], the authors introduced a novel Multi-Scale Deep Graph Convolutional Network (MS-DGCN) algorithm by incorporating multi-scale intra-class fine-to-coarse layers and multi-scale convolutional kernels. This approach addresses the challenge of obtaining multi-scale information in rotor-bearing systems. Collectively, these studies underscore the efficacy of applying single-sensor graphs to FD methods.

With the increasing complexity of engineering requirements, the demand for multi-sensor data processing has risen. In contrast with single-sensor data, multi-sensor data can furnish fault measurement information from diverse locations [16]. The authors of [17] combined multiple sensors to generate spatio-temporal graphs, thereby enhancing model performance by concurrently modeling sensors’ spatial and temporal dependencies. Another study [18] devised a multi-channel GCN, constructing corresponding undirected K-nearest-neighbor graphs for each sensor data. The methodologies mentioned above demonstrate the efficacy of GNNs in handling homogeneous multi-sensor data. Faced with challenges, including exponential multi-sensor data, an increasing number of actuators, and data-intensive algorithms, Wang et al. [19] noted the importance of heterogeneous information in aircraft systems. However, research on leveraging GNNs to learn the hidden topological relationships of heterogeneous mechanical equipment is still ongoing [20].

Given the stringent demands for safety, controllability, and repeatability in hypersonic aircraft, researchers have extensively explored ground semi-physical simulation technologies based on ERs [21]. We developed a Passive Torque Servo System (PTSS), a novel electric load simulator capable of emulating the hinge moments endured by ERs during flight. Consequently, multi-source sensor data delineating pertinent measurement information under various states, such as that from a PTSS or Control Surface (CS), can be acquired through the ER testing platform. Due to the challenges posed by homogeneous GNNs in showing interactions between different subsystems, our proposed solution is an end-to-end FD framework for heterogeneous mechanical equipment, named CEVAE-HGANN.

The specific contributions are as follows:

- A heterogeneous graph framework—CEVAE-HGANN—is proposed to deal with heterogeneous information in the ER test platform;
- An adjacency matrix construction method for HGANN is designed to control information flow using the Euclidean distance and Pearson coefficients, effectively relaying information between subsystems;
- A dimensional reduction method is proposed to attain efficient and robust features based on the Conditional Enhanced Variational Autoencoder (CEVAE).

## 2. Theoretical Background

### 2.1. Data Acquisition and Platform Description

The performance of the ER is crucial since it directly influences the overall functionality of the controlled system, particularly for hypersonic aircraft, and impacts control accuracy and hit rates [6]. For most mechanical devices, such as motor bearings, self-priming centrifugal pumps, and axial piston hydraulic pumps [22,23,24,25,26], FD typically entails the analysis of sensor data collected during actual operations. However, given the stringent safety, controllability, and repeatability requirements, along with the limitations in the quantity and quality of field tests for hypersonic aircraft, extensive research has been conducted on ground-based semi-physical simulation technology.

**Figure 1:**
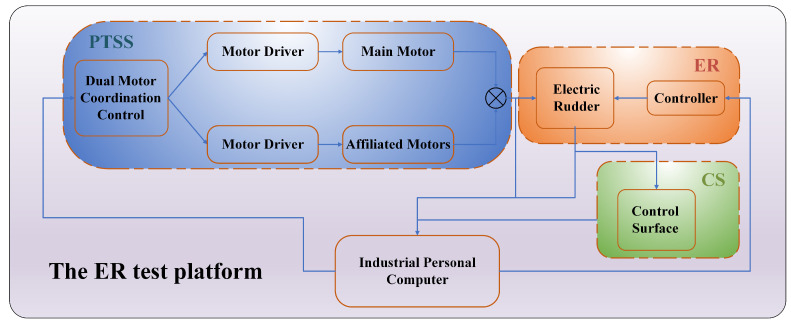
The ER test platform.

As shown in Figure 1, a test platform aimed at evaluating the ER has been developed. This platform comprises an ER used to convert electrical signals into mechanical actions, a Control Surface (CS), a signal acquisition unit, an industrial personal computer (IPC), a power supply unit, and a novel PTSS used to provide pre-set loads to the ER during testing procedures. The innovative PTSS employs a dual-motor cooperative control mechanism, encompassing a primary torque loading motor and a cooperative torque loading mechanism. When a large torque load is required, both the primary and cooperative motors are simultaneously driven in the same direction. For smaller torque loads, a single motor can be utilized, thereby offering significant flexibility. The cooperative control of dual motors helps to better mitigate disturbances, significantly enhancing the accuracy of the torque load application and the dynamic response speed. This setup ensures more accurate and efficient assessment and control of the ER’s performance, thereby contributing to the overall efficacy and safety of hypersonic aircraft operations.

### 2.2. Test Process and Data Details

The test platform simulates the process of the ER receiving electrical signals from the missile control system, operating the missile’s CS, and dynamically adjusting the flight path. The simulation test process is as follows: The ER moves based on position instructions provided by the IPC, causing the CS to displace. Subsequently, the CS’s position signals are measured in real time and fed back to the IPC, establishing a positional feedback loop. Concurrently, the IPC provides torque loading instructions to the PTSS, wherein the dual-motor coordination control drives the torque motor to output the torque. The load torque signals are then measured in real time and fed back to the IPC, creating a torque feedback loop. The IPC compares the expected torque values in the loading instructions with the actual measurements and computes the control amount. Then, it drives the torque motor via the dual-motor coordination control in the PTSS. This process adjusts the PTSS’s torque output to achieve and maintain the desired output.

The industrial personal computer serves as the nucleus of the entire ER test platform, providing the necessary infrastructure for operators to conduct repetitive testing, data mining, and experiments for FD in accordance with the ER working conditions. The proposed CEVAE-HGANN, along with other comparative methods, is implemented in the PyTorch framework and the Deep Graph Library Package [27]. This computational environment is hosted on a desktop workstation with internet connectivity, running on a Windows 10 operating system. The hardware supporting this setup includes an Intel (R) Core (TM) i9-9940X CPU operating at 3.30 GHz and a GeForce RTX 3090 GPU. The entire dataset encompasses a total of 20,000 samples, each encapsulating parameters representing the PTSS, ER, and CS subsystems at aligned moments. Specifically focusing on the ER, the object of FD, it has 10 classification labels. These include nine fault states (F1 to F9) and one normal state (NOR), implying that each classification label is represented by 2000 samples. The intricate details of the ER, PTSS, and CS are presented in Appendix A Table A1.

## 3. Proposed Method

This section outlines the steps of the CEVAE-HGANN model for ER-based FD. Throughout the FD process in CEVAE-HGANN, we categorize different nodes in the heterogeneous graph according to the subsystems and utilize meta-paths to describe the composite relationships between two subsystems. Specifically, to represent the heterogeneous relationships among the components, we define two meta-paths: CS-ER-CS and PTSS-ER-PTSS. Hence, we elucidate the CEVAE-HGANN model regarding adjacency matrix construction and node classification under different meta-paths.

### 3.1. Construction of Adjacency Matrices

**Figure 2:**
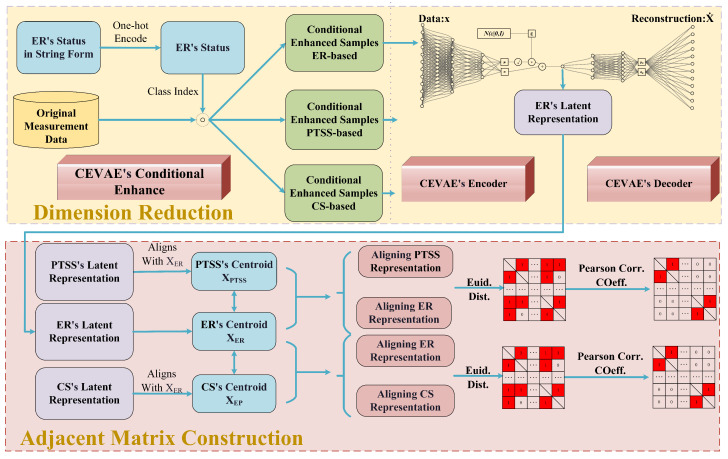
Construction of adjacency matrices.

Given that the sample distribution of each subsystem tends to become sparser with the increment in the sensor count, this undermines the referential validity of similarity measured by distance [28]. Concurrently, an increase in features also introduces substantial computational complexity. For each meta-path, we employ a strategy of initially performing dimensional reduction on the data and constructing adjacency matrices for relationship representation, as shown in Figure 2.

#### 3.1.1. CEVAE-Based Dimensional Reduction

To analyze the nonlinear high-dimensional ER data and accomplish downstream tasks, we employ a CEVAE for dimensional reduction to represent the overall operational state of the ER at a particular moment. Given the potential for complex nonlinear relationships between the state factors of the ER and the original features, which might not be readily observable directly from the original data, especially in a high-dimensional data space, we introduce the conditional vector c. A traditional CVAE [29], in its computational process, directly concatenates the conditional vector c as a new feature with the original features. Although this method integrates conditional information and original data as model inputs, the interaction manner is determined internally by the model, possibly necessitating a more complex model structure and additional training data to capture this interaction.

Inspired by the gating mechanism that retains or discards information to control feature flow, we first employ a one-hot encoding strategy to encode the conditional vector *c* and transform it into categorical indices, as shown in the top-left section of Figure 2. Subsequently, we perform a Hadamard product with the original features to obtain the interactive data. This approach amplifies the relevance between the conditional information and the original ER features, thus enhancing the representative capacity of the features.

We represent the interactively enhanced features with the sequence $x=[x_{1},x_{2},\ldots ,x_{T}]$, where *T* denotes the number of sensors describing the ER status. We introduce an uncertainty probabilistic distribution representation to capture the latent structural changes within the data. We posit that this sequence can be characterized by the latent variable *z* and presume that *z* adheres to a Gaussian distribution, that is:

(1) $$p\left(z\right)=N\left(0,I_{d*d}\right)$$

where *d* is the dimension and *I* is the identity matrix. The generative distribution is posited as follows:

(2) $$p_{\theta }\left(x|z\right)=N\left(\mu_{\theta }\left(z\right),\sum_{\theta }\left(z\right)\right)$$

Here, $\mu_{\theta }\left(z\right)$ and $\Sigma_{\theta }\left(z\right)$, respectively, denote the mean and covariance matrix of the data generated utilizing the latent variable *z*. Based on the assumptions mentioned above, we can derive $p\left(x\right)=\int p\left(x,z\right)\;dz=\int p\left(z\right)p_{\theta }\left(x|z\right)\;dz$. However, for high-dimensional data, p(x) is challenging to infer, which indirectly leads to the posterior probability distribution of the latent variable *z* as $p_{\theta }\left(z|x\right)=p\left(z\right)p_{\theta }\left(x|z\right)/p(x)$, which is also not inferable. Therefore, we employ a CVAE, specifically two fully connected layers, to generate the approximate posterior probability distribution $q_{\phi }\left(z|x\right)$ to approximate $p_{\theta }\left(z|x\right)$. However, since the Hadamard product utilized to obtain the interacted data introduces a more vital structural constraint [30], we simultaneously sample from the standard normal distribution ϵ, ensuring that the constraint is effectively considered during the training process. To more lucidly show the construction process and its practical implications, we selected an ER as the object for the investigation of the dimensional reduction method in the following sub-sections, as shown in the top-right section of Figure 2. The features of the PTSS and CS are reduced using the same method but are not shown in Figure 2.

The loss function, ELBO, of the CEVAE comprises a reconstruction loss computed using an L2 loss and a KL divergence, encouraging the CEVAE to learn a latent representation close to the prior distribution. The total loss function of the proposed dimensional reduction model can be expressed as:

(3) $$L_{\theta ,\phi }=-E_{q_{\phi }\left(z|x\right)}\left[\log p_{\theta }\left(x|z\right)\right]+\lambda_{\mathrm{kl}}\cdot \mathrm{KL}\left(q_{\phi }\left(z|x\right)\;\|\;p_{\theta }\left(z\right)\right)$$

Specifically, the first term represents the reconstruction loss, constructed using the L2 loss function, measuring the reconstruction error between the generated and original samples. The second term, $\mathrm{KL}(q_{⌀}\left(z|x\right)||p_{\theta }\left(z|x\right))$, is the KL divergence, evaluating the approximation effect, where $q_{⌀}\left(z|x\right)$ is the weight parameter for the loss term.

#### 3.1.2. Creating Correlation Adjacency Matrices

We employ a binary adjacency matrix to illustrate the temporal evolution of the relationships between the subsystems, serving as the edge information input for the HGANN. During the construction of the adjacency matrix, we use the Euclidean distance as the initial criterion for the correlation analysis between the subsystems at different moments. However, a notable consideration is that due to potentially different reference points and scales following dimensional reduction with varying weight parameters, the direct computation of the Euclidean distance between them lacks referential significance. Hence, we use the centroids of each subsystem as reference points, aligning the centroids of the PTSS and ER clusters to the origin to quantify their connectivity. We resolve this issue using singular-value decomposition (SVD) to find the optimal affine transformation parameters for the optimal alignment of the two data clusters. The process of data transformation is shown at the bottom of Figure 2. We first obtain the latent representations of the PTSS, ER, and CS based on the CEVAE. And then, we compute the centroids of the two subsystems $X_{CS}$ and $X_{ER}$ as $\left(x_{i},y_{i}\right)$ and $\left(x_{i}^{\prime },y_{i}^{\prime }\right)$, respectively, and translate the clusters to position their centroids at the origin, denoted as $X_{CS,center}$ and $X_{ER,center}$. Subsequently, we compute the covariance matrix *C* of the transformed CS and ER clusters. Then, we perform singular-value decomposition on the covariance matrix to obtain the optimal rotation angle *R* for data transformation.

(4) $$C=X_{\mathrm{CS},\mathrm{center}}^{T}\cdot X_{\mathrm{ER},\mathrm{center}}$$

(5) $$C=U\Sigma V^{T}$$

(6) $$R=V\cdot U^{T}$$

Subsequently, by utilizing the singular-value decomposition results, we calculate the rotation matrix and the scaling factor. We compute the translation matrix to align the CS data point set with the ER set. Then, we calculate the Euclidean distances at their respective timestamps. Finally, we determine the Euclidean distance between the ER and the transformed CS at time *t*, serving as the similarity measure at the current moment *t*. By setting a threshold for this similarity measure, whenever the absolute value of similarity is below the threshold, the corresponding rows and columns in the adjacency matrix are set to 0, indicating irrelevance. However, there are some limitations, given that the interactions between systems within the proposed ER testing platform are not instantaneous but exhibit certain time delays and dynamic behavior patterns. The Euclidean distance only considers the absolute distance between subsystems and is sensitive to outliers. In contrast, the Pearson correlation coefficient is less sensitive to outliers. When abnormalities in the ER trigger changes in the relationship intensity among other subsystems, the Pearson correlation coefficient can promptly capture such changes. We employ the Pearson coefficient as a secondary verification metric, which enhances the adjacency matrix’s stability and reduces unnecessary computational loads. Specifically, we first select an appropriate sliding time window size. We then calculate the Pearson correlation coefficient between the ER and CS feature vectors within each time window. The formula for deriving the Pearson correlation coefficient between the ER variable features, $x_{ER}$, and CS variable features, $y_{CS}$, is illustrated as follows:

(7) $$r\left(x,y\right)=\frac{\sum_{i=1}^{N}\left(x_{i}-\bar{x}\right)\left(y_{i}-\bar{y}\right)}{\sqrt{\sum_{i=1}^{N}{\left(x_{i}-\bar{x}\right)}^{2}}\sqrt{\sum_{i=1}^{N}{\left(y_{i}-\bar{y}\right)}^{2}}}$$

Next, the absolute value of the difference between the Pearson correlation coefficient $r_{t}$ and that of the previous time window $r_{t-1}$ is computed to represent the regional similarity *r*. By setting a threshold for this similarity measure, whenever the absolute value of *r* is below the threshold, the corresponding rows and columns in the original adjacency matrix are set to 0, indicating irrelevance.

Given that most of the nodes collected may not have direct connections to each other, constructing a sparse matrix enables focusing on highly relevant graph-structured data. In other words, by utilizing the revised adjacency matrix as the feature input for the HGANN, the control of information “flow” along the paths defined in the adjacency matrix is facilitated, providing an alternative perspective for efficiently analyzing the system’s overall performance.

### 3.2. HGANN-Based Model for Node Classification

**Figure 3:**
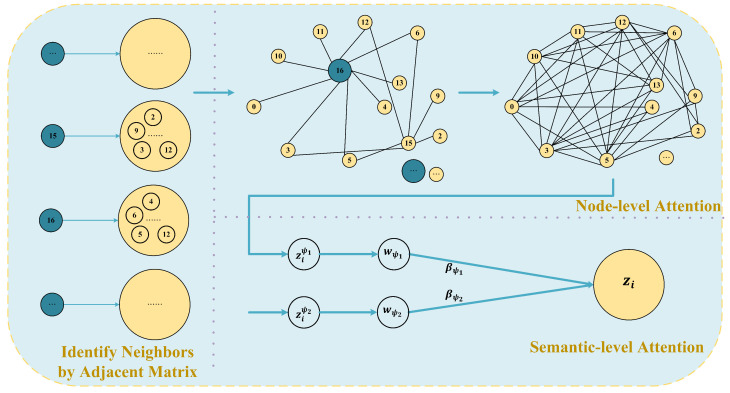
HGANN-based model for node classification.

In the proposed method, we employed the HGANN for classification, effectively capturing the features of graph information data from nodes and edges. Based on the procedures described in Section 3.1, we sequentially prepare heterogeneous graph data, establish heterogeneous graph embedding, and build the heterogeneous graph classification model. Initially, we vectorize the nodes and edges in the heterogeneous graph. Three node types are defined: PTSS, ER, and CS. We represent each node type’s respective node features in vector form and normalize them. Then, two adjacency matrices are obtained utilizing the method outlined in Section 3.1. These matrices represent the connection relationships among the different nodes and provide edge information for the HGANN. We divide the established heterogeneous graph embedding into node-level attention and semantic-level attention, as shown in Figure 3. We randomly extract a time window from the obtained adjacency matrices and demonstrate the neighboring nodes of the ER apparatus under the CS-ER-CS meta-path for two time windows (15, 16). For instance, the ER at moment 15 offers connectivity with the CS at moments 2, 5, 6, 9, and 12 in this time window.

We denote the node features as $h_{i}$. Initially, we perform a linear transformation on the input features of each type of node. Specifically, by setting the transformation matrix *W*, we unify the features of different node types and dimensions into a common feature space. This enhancement allows the model to capture and learn the complex interactions among the different nodes and edge types in the graph, thereby improving the model’s expressive capability. The transformed node features ${\left(h_{i}\right)}^{\prime }$ can be represented as:

(8) $$h_{i}^{\prime }=W*h_{i}$$

Next, based on the edge connectivity information described in Section 3.1.2, we use node-level attention to learn the neighbor weights, thereby distinguishing the importance of the neighbor nodes to the target node. Then, with semantic-level attention, we ascertain the contributions and importance of different meta-paths to FD. Specifically, in node-level attention learning, HGANN employs GAT (Graph Attention Network) layers to compute the attention weights between the target node *i* and its adjacent node *j* [31]. Given nodes *i* and *j* connected under a specified meta-path ψ, the node-level attention $\theta_{ij}^{\psi }$ can be represented as:

(9) $$\theta_{ij}^{\psi }={\mathrm{att}}_{\mathrm{node}}\left(h_{i}^{\prime },h_{j}^{\prime };\psi \right)$$

After obtaining the importance between nodes *i* and *j*, we normalize them using the softmax function to obtain the weight coefficient:

(10) $$\alpha_{ij}^{\psi }=\mathrm{softmax}\left(\theta_{ij}^{\psi }\right)=\frac{\exp(\mathrm{LeakyReLU}\left(a^{T}\left[h_{i}^{\prime }\|h_{j}^{\prime }\right]\right))}{\sum_{k\in N_{i}^{\psi }}\exp\left(\mathrm{LeakyReLU}\left(a^{T}\left[h_{i}^{\prime }\|h_{k}^{\prime }\right]\right)\right)}$$

where *k* represents the number of neighbors, || represents concatenation, and ${\left(\cdot \right)}^{T}$ represents transpose. These weights represent the importance of the neighbors to the target node based on a particular meta-path. Then, using these weights, we generate the feature of node *i* aggregated from the surrounding neighbor nodes to capture the complex relationship between the node and its neighbors:

(11) $$z_{i}^{\psi }=\sigma \left(\sum_{j\in N_{i}^{\psi }}\alpha_{ij}^{\psi }\cdot h_{j}^{\prime }\right)$$

Here, each node in the heterogeneous graph contains multiple semantic information. The node embeddings obtained by the above node-level attention only reflect node information from one aspect. To learn more comprehensive node embeddings, we need to employ semantic-level attention to reveal multiple semantics through meta-paths. In semantic-level attention, for the weight $z_{i}^{\psi }$ of a certain node and its surrounding neighbors, first, pass through a fully connected layer and apply the tanh activation function, and then perform the dot product with a learnable parameter *q*, thus obtaining the scalar corresponding to a certain node under a meta-path. Then, sum and average the scalars of each node under this meta-path to obtain the importance of each meta-path $w_{\psi_{i}}$, which can be expressed as:

(12) $$w_{\psi_{i}}=\frac{1}{\left|V\right|}\sum_{i\in V}q^{T}\cdot \tanh\left(W\cdot z_{i}^{\psi }+b\right)$$

where *W* is the weight matrix, *b* is the bias vector, and *q* is the semantic attention vector. Following this, the softmax function is utilized to normalize the importance $w_{\psi_{i}}$ of each meta-path, obtaining the weight $\beta_{\psi_{i}}$ of meta-path $\psi_{i}$:

(13) $$\beta_{\psi_{i}}=\frac{\exp(w_{\psi_{i}})}{\sum_{i=1}^{P}\exp\left(w_{\psi_{i}}\right)}$$

Through the learned weights $\beta_{\psi_{i}}$, the final node embedding *Z* is obtained and can be expressed as:

(14) $$Z=\sum_{i=1}^{P}\beta_{\psi_{i}}\cdot z_{i}^{\psi }$$

Lastly, for the node classification task, the cross-entropy loss of the model’s predictions on all labeled nodes is minimized to train the model and obtain the optimal parameters.

## 4. Experimental Results and Analysis

The data collected from the ER test platform were fed into the CEVAE-HGANN model for training. Here, we summarize the hyperparameters involved in the CEVAE-HGANN process, including the rationale behind the selection of some of these parameters. The evaluation metrics and experimental results are presented in the subsequent subsections. Then, we analyze the outcomes of the comparative and ablation experiments, highlighting the potential limitations of the proposed model.

### 4.1. Acquisition Method of Key Parameters

#### 4.1.1. Parameter Setting for the CEVAE-HGANN

For the proposed CEVAE model, the learning rate was set to 0.001, and the weight $\lambda_{kl}$ of the KL divergence in the loss was set to 0.2. For the proposed HGANN model, the learning rate was set to 0.01, the regularization parameter was set to 0.001, random initialization of parameters was used, and the model was optimized using the Adam optimizer [32] with an early stopping patience of 100. For the attention mechanism utilized in the HGANN, the number of attention heads *K* was set to 8, the dimension of the semantic-level attention vector *q* was set to 128, and the attention dropout was set to 0.6. All experiments were performed with tenfold cross-validation to obtain relatively reliable evaluation results.

#### 4.1.2. Setting the Size of the Time Window and the Threshold

**Figure 4:**
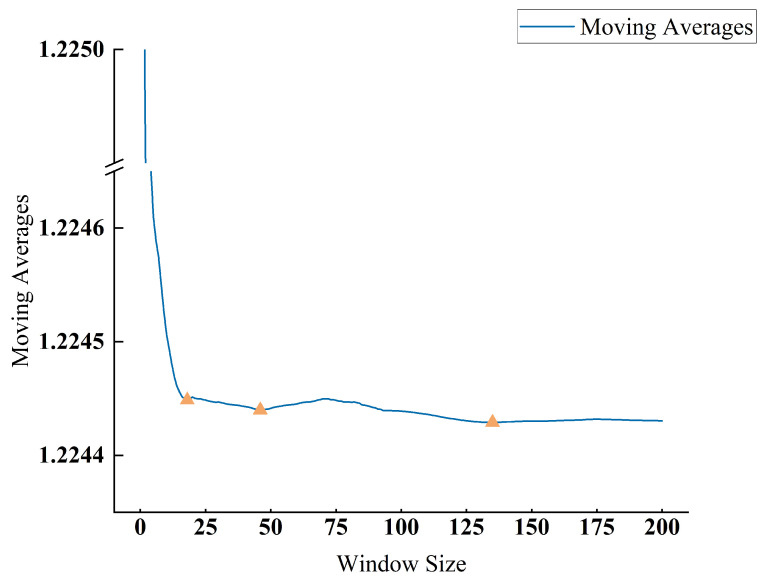
Selecting the window size.

The proposed model requires the determination of some key parameters alongside the hyperparameters that need empirical and temporal trials. These key parameters include the threshold values utilized in constructing adjacency matrices and the size of the time window. We utilized the method in [33] to determine the suitable window size for the given ER sequence. Specifically, the moving average of the time series was computed for a given window size. The moving average was derived by computing the average value of each continuous sub-sequence in the time series. For each window size’s moving average, the absolute distance of these moving averages from their overall average was computed, denoted as the moving-dist meta-time series. The optimal window size was determined by locating the position of the first valley in the moving-dist meta-time series. As shown in Figure 4, a window size of 18 was deemed suitable.

**Figure 5:**
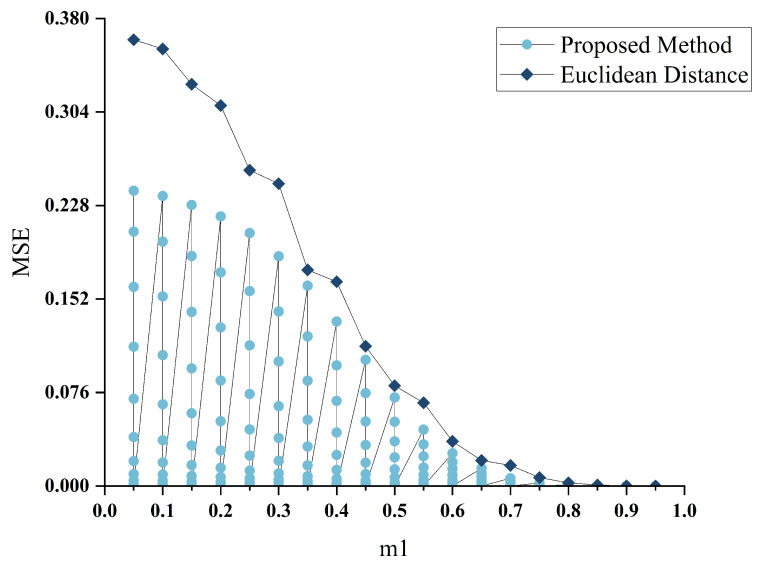
Setting the threshold for $m_{1}$.

**Figure 6:**
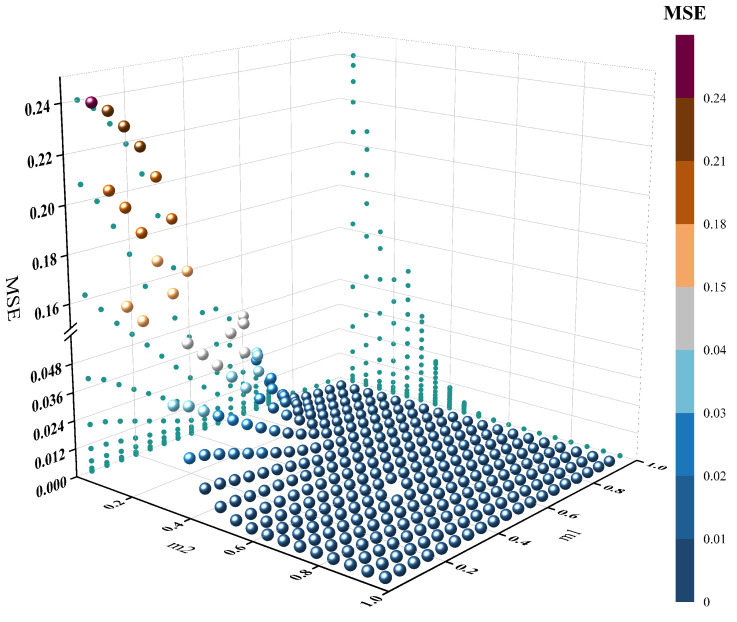
Setting the thresholds for $m_{1}$ and $m_{2}$.

The described scenario entails hypersonic aircraft undergoing testing, during which structural vibrations, motion-induced noise, and electromagnetic interference may disrupt the test parameters. The adjacency matrix, representing the connectivity relationships between subsystems, is crucial to the model’s robustness. An overly sensitive adjacency matrix could offer better stability but might forfeit some vital information for the HGANN model. The optimal threshold for the adjacency matrix was determined by analyzing the Mean Squared Error (MSE) of the adjacency matrices before and after adding standard Gaussian noise under varied threshold values, as shown in Figure 5. As the sensitivity values increased, the MSE transitioned from a steep decline to a more gradual one. To balance the adjacency matrix’s information retrieval capability and stability, the saddle points *x* and *y* were selected as the sensitivity values, with $m_{1}$ and $m_{2}$ being 0.63 and 0.58, respectively. A simple visualization elucidates the selection method, with the x-axis in Figure 6 representing the sensitivity used in calculating the Euclidean distance and the y-axis representing the MSE under two adjacency matrix construction methods. Notably, in the proposed method’s curve, one $m_{1}$ corresponds to multiple $m_{2}$ values, where $m_{2}$ represents the sensitivity used in calculating the Pearson coefficients. Figure 6 also demonstrates the robust stability of the proposed adjacency matrix construction model, which, to some extent, substantiates the necessity of using Pearson coefficients as a secondary verification of the connectivity.

### 4.2. Experimental Results and Analysis

#### 4.2.1. Evaluation Metrics

The performance of the CEVAE-HGANN model was evaluated using multi-classification metrics. The employed evaluation metrics included the Accuracy, Precision, TPR, TNR, and $F_{1}$-Score. The formulas for these metrics are as follows:

(15) $$\mathrm{Accuracy}=\frac{TP+TN}{TP+TN+FP+FN}$$

(16) $$\mathrm{Precision}=\frac{TP}{TP+FP}$$

(17) $$\mathrm{TPR}=\frac{TP}{TP+FN}$$

(18) $$\mathrm{TNR}=\frac{TN}{TN+FP}$$

(19) $$F_{1}-\mathrm{Score}=\frac{2\times \mathrm{Precision}\times \mathrm{TPR}}{\mathrm{Precision}+\mathrm{TPR}}$$

where TP, FP, FN, and TN are, respectively, the number of true positives, false positives, false negatives, and true negatives. In this study, the NOR sample is considered positive, whereas the remaining samples are considered negative.

#### 4.2.2. Performance Comparison of Dimensional Reduction Methods

For dimensional reduction, Principal Component Analysis (PCA) [34] and its variant Kernel PCA (KPCA) [35] were utilized as the benchmarks since they are widely used linear and nonlinear dimensional reduction methods in FD of mechanical equipment. In the comparative experiment, these two methods were used as non-generative dimensional reduction methods for comparison with the proposed method. Additionally, an autoencoder [36] and the classical VAE [37], with a similar architecture to the proposed method, were included in the comparative experiment as generative dimensional reduction methods.

**Figure 7:**
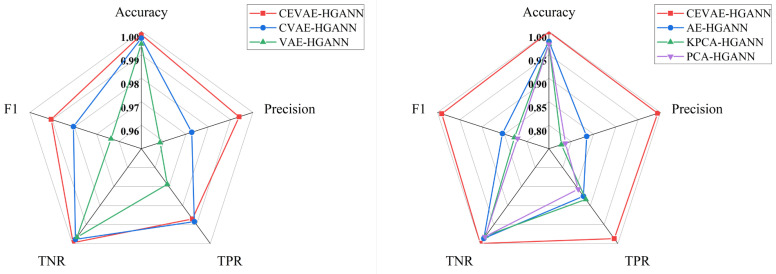
Performance comparison of dimensionality reduction methods.

The information in Table 1 indicates that the impact of dimensional reduction on the edge number was not definitive. However, a noteworthy increase in the $F_{1}$-Score, ranging between 0.06% and 0.91%, can be observed. Further, the graphical representation elucidates the comparative advantage of generative dimensional reduction methods over non-generative ones for the ER subsystem, as shown in Figure 7. Particularly, CEVAE emerged as the most effective method among the evaluated techniques. The performance metrics exhibited substantial improvements, where the Accuracy increased from 0.15% to 2.61%, Precision increased from 2.12% to 21.61%, TPR increased from 0.14% to 13.06%, TNR increased from 0.17% to 1.94%, and $F_{1}$-Score increased from 1.00% to 17.01%. These metrics underscore the positive impact of effective dimensional reduction on the construction of adjacency matrices. In addition, as seen in Table 2, the edge number was reduced from 97.962% to 98.178% when employing the Pearson correlation coefficient for secondary verification. However, despite this significant reduction, the $F_{1}$-Score increased from 0.50% to 0.89%. This indicates that a large proportion of the neighboring nodes in the adjacency matrix might be redundant or possibly noise-inducing, which could adversely affect the weight analysis of the target nodes. The Pearson correlation coefficient, serving as a secondary verification method, appeared to efficiently filter out these redundant or less informative edges, thereby refining the structure of the adjacency matrix. A well-structured adjacency matrix is instrumental in efficiently directing the “flow” of information along defined pathways within the network.

#### 4.2.3. Performance Comparison of Classification Methods

**Figure 8:**
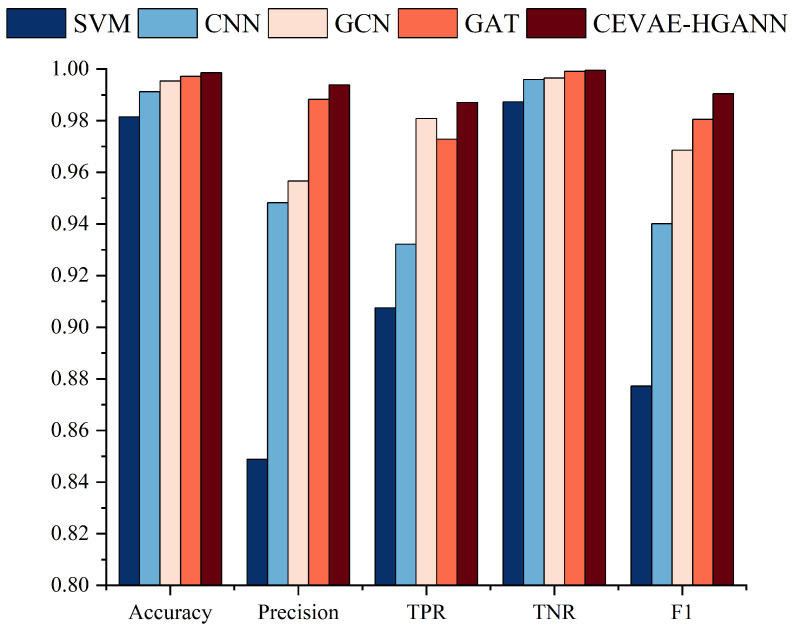
Performance comparison of classification methods.

For classification, initially, methods like BP (Back-Propagation), SVMs (Support Vector Machines), and CNNs (Convolutional Neural Networks), with their respective parameters obtained from the above-referenced papers, were selected as the FD methods based on Euclidean data. Subsequently, more representative methods in the graph data domain, GCN and GAT, were selected for comparison. Figure 8 shows that all FD methodologies achieved high accuracy. This can be attributed to the evident fault signatures in the ER’s fault scenarios, enabling models to easily recognize anomalous states and achieve high accuracy. For instance, although the CS angle deflection feature may help distinguish between normal and fault statuses, it cannot differentiate between various fault statuses. However, a discernible disparity in precision among the different methodologies was observed. The precision of the SVM- and CNN-based classification methods was significantly inferior to that of the graph data-based methods, elucidating that graph-based methodologies can more adeptly accommodate the intricate interactions within the ER, as opposed to being merely confined to local features. For the graph data-based methods, as shown in Table 1, the increases in the Accuracy (0.15–1.72%), Precision (0.56–14.50%), TNR (0.04–1.224%), and $F_{1}$-Score (1.00–11.322%) are evidence of the superior capability of the HGANN to capture the complex interactions among the ER, PTSS, and CS, thereby resulting in higher precision in identifying specific fault states.

### 4.3. Discussion and Limitations

By conducting a series of ablation and comparative experiments and using the final FD results as the evaluation metric, we evaluated the performance of various dimensional reduction and classification methods on the ER. This substantiated the superiority of the CEVAE-HGANN method over other methods and elucidated the impact of each step (dimensional reduction and feature extraction) on the overall performance of the method.

From the experimental results and analysis, we found that both standard machine learning and deep learning methods, as well as our proposed graph neural network-based approach, achieved high $F_{1}$-Scores. However, in practical applications, the ER, as a directional control component in hypersonic aircraft, typically requires at least four identical ERs to form a complete Electrical Rudder Servo System (ERSS) for control. This implies that any minor discrepancies in misdiagnosis rates will be extensively magnified in the final ERSS fault diagnosis process. The benefits of reducing the misdiagnosis rates will massively accumulate within the system. Moreover, the interaction among components during the loading process cannot be ignored. The proposed model needs to build relationships between different ERs; thus, the CEVAE-HGANN model demonstrates promising potential.

The limitations of CEVAE-HGANN can be summarized as follows: (1) Bias in adjacency matrix construction: Utilizing the Pearson correlation coefficient for secondary verification in adjacency matrix construction, although effective in reducing data redundancy by around 97.962–98.178%, introduces a certain level of subjective bias. This bias arises from the sole reliance on similarity metrics to establish connections, which may affect node embedding. (2) Threshold selection in adjacency matrix construction: The method employed for threshold selection in the adjacency matrix construction process involves visualization to ascertain a rough interval, followed by iteration, which inevitably entails subjectivity.

## 5. Conclusions

To better diagnose the ER, we present CEVAE-HGANN, a novel FD method, whose novelties can be summarized as follows: (1) To address the shortcomings found in conventional Euclidean data-driven FD methods, we introduce a novel method, CEVAE-HGANN, aimed at heterogeneous information. This method tackles FD challenges across diverse information from subsystems, moving beyond node-level feature dependency. It underscores the interconnections among various subsystems, providing a fresh lens for evaluating a system’s overall system performance. The efficacy of CEVAE-HGANN was assessed on a dataset from the testing platform, with improvements in the Accuracy (0.15–1.72%), Precision (0.56–14.50%), TNR (0.04–1.224%), and $F_{1}$-Score (1.00–11.322%), indicating superior results compared to the baseline model. (2) For effective information transmission among subsystems within the HGANN, we suggest a new adjacency matrix formulation technique to outline inter-system connections. This technique controls information flow along the designated paths in the adjacency matrix, facilitating a streamlined analysis of a system’s overall performance within a heterogeneous graph. The $F_{1}$-Score improved by 0.50% to 0.89% using the proposed construction method. (3) To achieve robust and efficient features with reduced computational overhead, we propose a dimensional reduction technique based on the CEVAE and employ it to formulate the adjacency matrix. The performance metrics exhibited substantial improvements, including increases in the Accuracy of 0.15% to 2.61%, Precision of 2.12% to 21.61%, TPR of 0.14% to 13.06%, TNR of 0.17% to 1.94%, and $F_{1}$-Score of 1.00% to 17.01%.

The focuses of future research can be summarized as follows: (1) Bias in adjacency matrix construction: Exploring more generalized methods for adjacency matrix construction that do not solely rely on similarity metrics but also consider other structural or contextual information may mitigate this bias. (2) Threshold selection in adjacency matrix construction: Automated or algorithm-driven threshold selection methods that can adaptively determine optimal values based on the data characteristics may offer a more objective and effective approach. (3) Performance of the HGANN in heterogeneous systems: Exploring strategies like time decay or other mechanisms for linking dynamic graphs at different time points might enhance the HGANN’s efficiency, particularly in dynamically evolving systems.

## Figures and Tables

**Table 1:** Performance comparison of classification methods.

Method	SVM	CNN	GCN	GAT	CEVAE-HGANN
Accuracy	0.98145	0.99125	0.99535	0.99715	0.99860
Precision	0.84881	0.94824	0.95661	0.98825	0.99382
TPR	0.90753	0.93216	0.98084	0.97279	0.98704
TNR	0.98727	0.99595	0.99649	0.99908	0.99951
F1-Score	0.87719	0.94013	0.96857	0.98046	0.99042

**Table 2:** Graph quality when constructing meta-path graphs based on the different methods.

No Red.	CEVAE-Based Red.	Euclid. Dist.	Pearson Corr. Coeff.	Edge Number PTSS-ER-PTSS	Edge Number CS-ER-CS	*F*_1_-Score
✓	-	✓	-	26,164,687	28,158,692	98.092%
✓	-	✓	✓	525,609	522,760	98.142%
-	✓	✓	-	25,738,246	28,696,637	98.148%
-	✓	✓	✓	473,718	573,737	99.048%

## Data Availability

Data are unavailable due to privacy or ethical restrictions.

## Appendix A

**Table A1:** The parameters of the dataset.

Subsystem	Parameter	Details
PTSS: Principally simulates and compensates for external loads and is responsible for providing pre-set loads to the ER during testing procedures.	Torque loading	The action of applying a specified torque to test the performance of the ER
	Hysteresis characteristics	The lag observed between the output and the input commands upon receiving a torque loading instruction is indicative of the system’s response time and possibly the inherent hysteresis.
	Torque loading frequency	The rate at which torque loading is applied.
	Loading transition response	The behavior of a system when transitioning from one level of loading to another.
	Maximum drive force (outward)	The highest amount of force that can be transmitted in an outward direction by the system.
	Maximum drive force (inward)	The highest amount of force that can be transmitted in an inward direction by the system.
	Hysteresis characteristics	The property of the system in which the response to a change in input depends on the current and past states, indicating a lag or delay in the response.
	No-load bandwidth	The frequency range over which the system can operate effectively without any load.
ER: Primarily responsible for converting electrical signals into mechanical actions.	Load bandwidth	The frequency range over which the system can operate effectively with a specified load.
	Overshoot in forward step response	The amount by which a system’s response to a forward step input exceeds its final steady-state value.
	Overshoot in reverse step response	The amount by which a system’s response to a reverse step input exceeds its final steady-state value.
	Steady-state error for positive control signal	The difference between the final output and the desired output for a positive control signal once the system has reached a steady state.
	Steady-state error for negative control signal	The difference between the final output and the desired output for a negative control signal once the system has reached a steady state.
	No-load response time	The time it takes for the system to respond to an input under no-load conditions.
	Rise time for positive control signal	The time it takes for the response to a positive control signal to rise from a specified lower percentage to a specified higher percentage of its final value.
	Rise time for negative control signal	The time it takes for the response to a negative control signal to rise from a specified lower percentage to a specified higher percentage of its final value.
CS: The actual control section of the aircraft, responsible for the aircraft’s maneuvering.	Angle of rotation	Given the control signal of the servo motor, the maximum angle over which the servo can rotate.
	Maximum travel (outward)	The maximum angle the system can move in the outward direction up to a specified limit.
	Maximum travel (inward)	The maximum angle the system can move in the inward direction up to a specified limit.
	Dead zone (outward)	After giving the outward command, the threshold point where the command starts to take effect and initiate rotation.
	Dead zone (inward)	After giving the inward command, the threshold point where the command starts to take effect and initiate rotation.
